# Inequalities in the prevalence recording of 205 chronic conditions recorded in primary and secondary care for 12 million patients in the English National Health Service

**DOI:** 10.1186/s12916-024-03767-4

**Published:** 2024-12-02

**Authors:** Shaolin Wang, Yiu-Shing Lau, Matt Sutton, Michael Anderson, Christodoulos Kypridemos, Anna Head, Ben Barr, Richard Cookson, Chris Bentley, Laura Anselmi

**Affiliations:** 1 Health Organization, Policy and Economics, University of Manchester, Manchester, UK; 2 Department of Public Health, Policy and Systems, University of Liverpool, Liverpool, UK; 3 Centre for Health Economics, University of York, York, UK; 4 Independent Population Health Consultant, Sheffield, UK

**Keywords:** Inequality, Chronic conditions, Primary care, Secondary care, Diagnostic recording

## Abstract

**Background:**

Understanding the prevalence of diseases and where it is detected and recorded in healthcare settings is important for planning effective prevention and care provision. We examined inequalities in the prevalence of 205 chronic conditions and in the care setting where the related diagnoses were recorded in the English National Health Service.

**Methods:**

We used data from the Clinical Practice Research Datalink Aurum linked with Hospital Episode Statistics for 12.8 million patients registered with 1406 general practices in 2018. We mapped diagnoses recorded in primary and secondary care in the previous 12 years. We used linear regressions to assess associations of ethnicity, deprivation, and general practice with a diagnosis being recorded in primary care only, secondary care only, or both settings.

**Results:**

72.65% of patients had at least one diagnosis recorded in any care setting. Most diagnoses were reported only in primary care (62.56%) and a minority only in secondary care (15.24%) or in both settings (22.18%). Black (− 0.08 percentage points (pp)), Asian (− 0.08 pp), mixed (− 0.13 pp), and other ethnicity patients (− 0.31 pp) were less likely than White patients to have a condition recorded. Patients in most deprived areas were 0.27 pp more likely to have a condition recorded (+ 0.07 pp in secondary care only, + 0.10 pp in both primary and secondary care, and + 0.10 pp in primary care only). Differences in prevalence by ethnicity were driven by diagnostic recording in primary care. Higher recording of diagnoses in more deprived areas was consistent across care settings. There were large differences in prevalence and diagnostic recording between general practices after adjusting for patient characteristics.

**Conclusions:**

Linked primary and secondary care records support the identification of disease prevalence more comprehensively. There are inequalities in the prevalence and setting of diagnostic recording by ethnicity, deprivation, and providers on average across conditions. Further research should examine inequalities for each specific condition and whether they reflect also differences in access or recording as well as disease burden. Improving recording where needed and making national linked records accessible for research are key to understanding and reducing inequalities in disease prevention and management.

**Supplementary Information:**

The online version contains supplementary material available at 10.1186/s12916-024-03767-4.

## Background

Diagnoses recorded in routinely collected data, including patient electronic health records, are increasingly used in public health research to estimate disease prevalence and burden [1]. This information is key to identifying patients at risk, planning and organising prevention and care, allocating resources, and setting research priorities. However, studies mapping disease prevalence comprehensively are limited [2]. Some have investigated associations of disease prevalence with population sociodemographic characteristics [3], and others investigated how recorded prevalence varies by setting [1]. Most studies examining differences in diagnostic recording between care settings are focused on specific diseases. There is a lack of evidence on the association between prevalence and diagnoses recorded by setting, and sociodemographic factors across a comprehensive selection of common conditions. This information is essential to tackle health inequalities through health service planning and provision.

Existing studies on diagnostic recording have adopted disparate case definitions and methodologies and have used different study samples, preventing comparability and generalisation across studies. Studies looking at single diseases highlighted discrepancies in recording between primary and secondary care records and attributed differences to diseases’ clinical care pathways, coding systems and practices [4–6], and how diseases are diagnosed, prevented, and managed for specific population groups in different care settings [7–10].

Two studies looking at a broader range of diseases found that the prevalence of diagnosis recording was higher when identified using primary care versus secondary care data. MacRae and colleagues [1] systematically compared the sources of diagnosis recording for a broad range of diseases in primary and secondary care and found that the prevalence of recording was higher in primary care for 37 out of 47 chronic conditions in Wales. Furthermore, Crooks et al. [11] examined the number of comorbidities identified using the Charlson Comorbidity Index and found approximately three times more comorbidities identified in primary care records than in secondary care records. A recent review highlighted that the estimated prevalence of multimorbidity was significantly higher in studies that included a larger number of conditions [12].

Recent studies have developed methods to map the prevalence of several hundred diseases in different care settings and administrative records for a representative sample of the English population. Notably, Kuan et al. [2] developed case definitions for 308 acute and chronic conditions and produced the first chronological map of human health using linked primary and secondary care records for 4 million individuals. Prevalence estimates were stratified by demographic characteristics (age, sex, and ethnicity). Head et al. [3] focused on a subset of 209 conditions classified as chronic conditions. Using primary care records for 1 million adults, the study characterised the prevalence of multimorbidity by age, sex, deprivation, and region. However, neither of these studies examined differences and inequalities in diagnosis recording between primary and secondary care settings.

This study applies the methods developed by Kuan et al. [2] and Head et al. [3] to undertake the first assessment of inequality in disease prevalence and diagnosis recording between care settings by ethnicity, deprivation, and general practice in England. In total, we examine diagnosis recording for 205 chronic conditions identifiable in primary and secondary care settings, using linked primary and secondary care records of 12.8 million patients in England.

## Methods

### Data sources and study population

We used anonymised patient-level longitudinal primary care data from the Clinical Practice Research Datalink (CPRD) Aurum (database version June 2021) linked to the Hospital Episode Statistics (HES) data (set 21).

CPRD Aurum is one of the largest primary care databases globally, comprising electronic health records for 1478 consenting general practices in England and over 14 million active (alive and currently registered) patients (23% of the population of England) at the time of data extraction (June 2021). CPRD is broadly representative of the English primary care population [13] and has been extensively used for epidemiological research. It holds data on demographics (patient’s age, sex, and ethnicity), diagnoses coded using clinical codes in EMIS® software system, test results, referrals, prescriptions, practice registration period, and pseudonymised practice ID.

HES is a large hospital database that has been collecting administrative data from NHS hospitals in England for remuneration purposes since 1997. HES Admitted Patient Care data include demographic information (age, sex, and ethnicity), hospital episode information (admission and discharge dates), diagnoses coded using the International Classification of Diseases version 10 (ICD-10), and procedures undertaken coded using the UK Office of Population, Censuses and Surveys Classification (OPCS) 4.6 [14].

We also obtained data on the 2019 English Index of Multiple Deprivation (IMD) [15] quintile of each patient’s area of residence (32,844 Lower Super Output Area, LSOA, in 2019, each including on average 1500 individuals) and death registration data from the Office for National Statistics.

We identified 13,135,862 eligible patients in CPRD Aurum registered with one of 1478 general practices in England on 1 April 2018 and whose records met research standards set by CPRD. We retained 12,755,868 patients of all ages from 1406 GP practices for the study (Additional File S1). From linked records, we identified ethnicity for 88.33% of individuals (Additional File S2 [16, 17]). We classified patients into 14 ethnic groups, including a category grouping “unknown”.

The protocol was approved by the CPRD Independent Scientific Advisory Committee (protocol 21–000693).

### Diagnoses and population characteristics

We mapped physical and mental health chronic conditions identifiable in primary and secondary care data using the phenotyping algorithms and codelists developed by Kuan et al. [2] as published on the CALIBER Portal [18] and adapted to CPRD Aurum by Head et al. [19]. In total, we mapped 205 out of 209 chronic physical and mental health chronic conditions used by Head et al. 2020. We did not map four chronic conditions (low HDL-C, raised LDL-C, raised total cholesterol, and raised triglycerides) as they are almost exclusively managed in primary care and, therefore, not suitable to analyse diagnostic recording practices in secondary care. We used any pre-existing and new diagnoses recorded between 1 April 2006 and 31 March 2018. We chose this period as it started 2 years after the launch of the Quality and Outcomes Framework programme in 2004, which impacted diagnostic recording in primary care [20]. We identified chronic conditions recorded during the period, the setting of the recording, and the date of the first recording. We classified diagnosed cases into three mutually exclusive categories based on the recording setting: primary care only, secondary care only, and both.

We used information on patient characteristics, including 10-year age groups, sex, ethnic groups, quintile of the IMD 2019 of the patient’s area of residence, and whether the patient was newly registered with the GP practice within the last year (see Additional File Table S1).

### Statistical analysis

First, we calculated the prevalence of each of the 205 chronic conditions, in total and by the care setting of diagnosis recording, among the registered population.

Second, we assessed the association of ethnicity, deprivation, and general practice with the setting where the diagnosis is recorded. We constructed an individual-condition level dataset. To obtain a more manageable dataset, we used a 50% random sample of patients stratified by age, gender, ethnicity, deprivation, and practice to maintain the relative size and patient composition of each practice [21]. We assessed associations for 194 conditions, excluding 11 conditions with 90% of diagnosed cases recorded only in primary care (i.e. acne, seborrheic dermatitis, rosacea, alopecia areata, scleritis and episcleritis, tinnitus, allergic and chronic rhinitis, vitiligo, dermatitis, vitamin B_12_ deficiency anaemia, and folate deficiency anaemia). These were mainly skin or immunological conditions that are exclusively treated in primary or outpatient care settings. For each observation, we used information on the setting of diagnostic recording, patient characteristics, a set of binary indicators for each of the remaining 194 conditions, and the practice of registration.

We estimated two sets of multivariable regressions. Each set included three regressions, one for each outcome indicating whether for each patient a given condition was recorded: (i) only in primary care, (ii) only in secondary care, and (iii) in both primary and secondary care settings. The first set included five ethnic categories, and the second set included five deprivation quintiles, with an additional category each for patients with missing or unknown ethnicity or deprivation respectively. We included controls for sex interacted with 10-year age bands, patients newly registered in the practice to account for potential under-ascertainment where an individual had recently moved practice, and binary indicators for each of 194 chronic conditions. We included GP practice fixed effects to account for systematic differences in diagnostic patterns across practices.

As a sensitivity analysis, we re-estimated two sets of regressions: first without practice fixed effects and second excluding pre-existing diagnoses recorded before the start of the study period (1 April 2006) to focus exclusively on the reporting of new diagnoses. We also used 14 low level ethnic categories instead of five.

We examined the distribution of the coefficients on the practice fixed effects, controlling for all observable patient-level characteristics, to show the remaining unexplained variation across practices.

We estimated linear probability models with standard errors clustered at the patient level. The analysis was carried out using Stata/MP 17.0.

## Results

Among the 12,755,868 patients, 9,267,558 (72.65%) had at least one chronic condition recorded in the linked data between 2006 and 2018, constituting 35,696,499 recorded diagnoses. Chronic conditions were predominantly recorded in primary care, with 30,249,675 (84.74% of diagnoses) recorded in primary care and 13,359,987 (37.43%) in secondary care.

There was a disparity in recording between care settings. 22,336,512 (73.84%) of diagnoses recorded in primary care were not recorded in secondary care. Among the diagnoses recorded in secondary care, 5,446,824 (40.77%) were not recorded in primary care, and 3,259,581 (24.40%) were subsequently recorded in primary care. In total, 62.57% of diagnoses were recorded only in primary care, 15.26% only in secondary care, and 22.17% in both primary and secondary care. Ninety-one conditions were predominantly recorded in primary care, 59 in secondary care, and 55 in both care settings (Additional File Table S2).

**Fig. 1:**
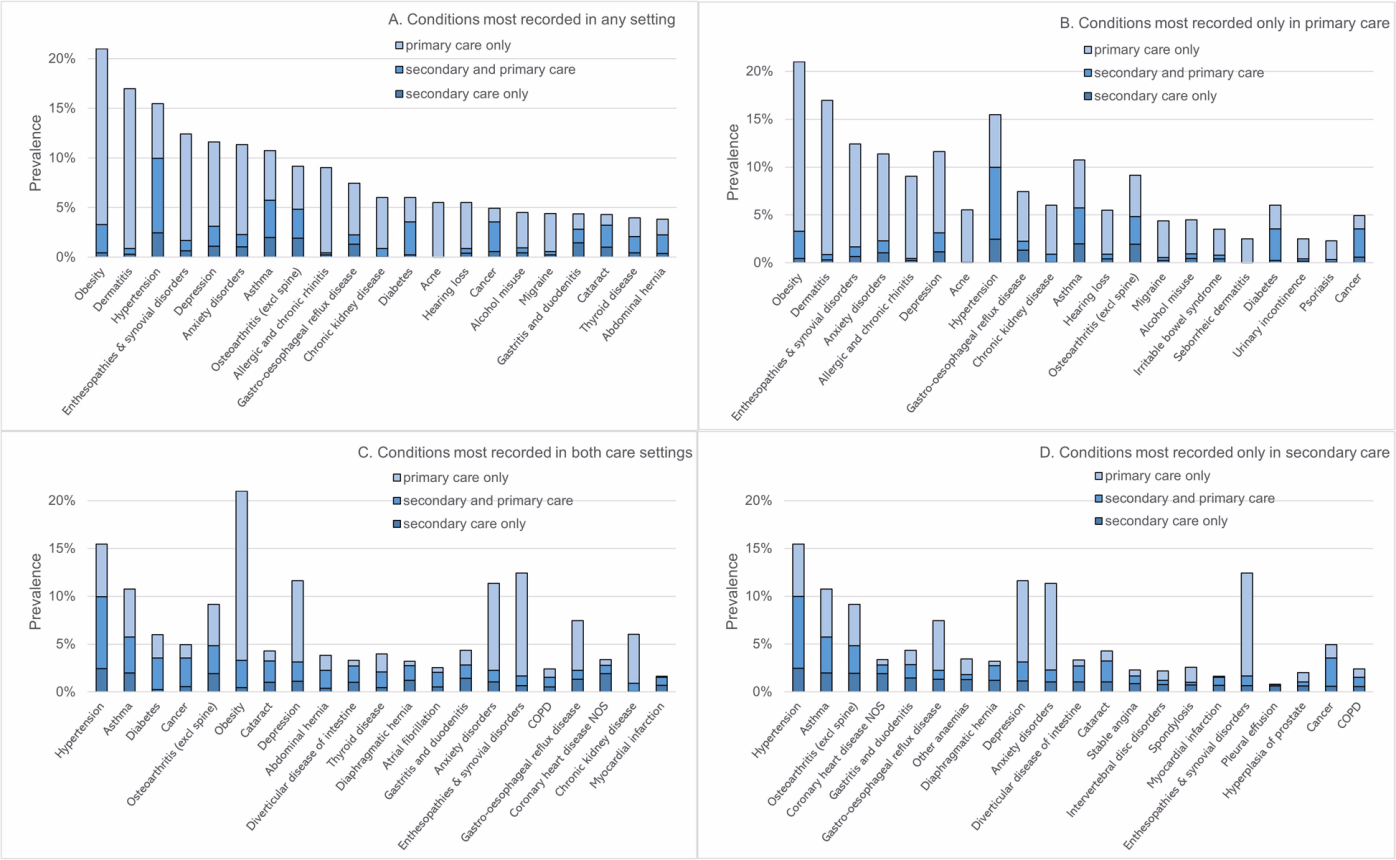
Prevalence of recorded diagnoses by setting for 20 most prevalent conditions

The most prevalent conditions across patients were obesity (prevalence: 20.99%), dermatitis (16.97%), hypertension (15.46%), enthesopathies and synovial disorders (12.42%), depression (11.63%), anxiety disorders (11.36%), and asthma (10.75%) (Fig. 1A; Additional File Table S2). However, prevalence was different when considering records from specific care settings. Of the 205 conditions, obesity (17.68%) and dermatitis (16.08%) had the highest prevalence when considering diagnoses reported in primary care only, while hypertension was the most prevalent when considering diagnoses in secondary care only (2.46%) and in both settings (7.52%). Other conditions often reported in primary care only included enthesopathies and synovial disorders (prevalence in primary care only: 10.74%), anxiety disorders (9.07%), and depression (8.48%) (Fig. 1B). Conditions most reported in both primary and secondary care included asthma (prevalence in both settings: 3.76%), diabetes (3.29%), cancer (2.98%), osteoarthritis (excl. spine) (2.89%), and obesity (2.85%) (Fig. 1C), and most reported in secondary care only included asthma (prevalence in secondary care only: 1.98%), osteoarthritis (1.94%), and coronary heart disease (1.91%) (Fig. 1D).

Breakdown by the recording setting may reflect where conditions require treatment (Additional File Table S2). Three fifths of diagnosed cancer (60.30%) and atrial fibrillation (60.11%) cases were recorded in both primary and secondary care. More than half of pleural effusion (79.29%) and coronary heart disease (56.51%) cases were recorded in secondary care only. The vast majority of acne (99.59%), dermatitis (95.10%), and allergic and chronic rhinitis (94.78%) cases were recorded in primary care only.

**Fig. 2:**
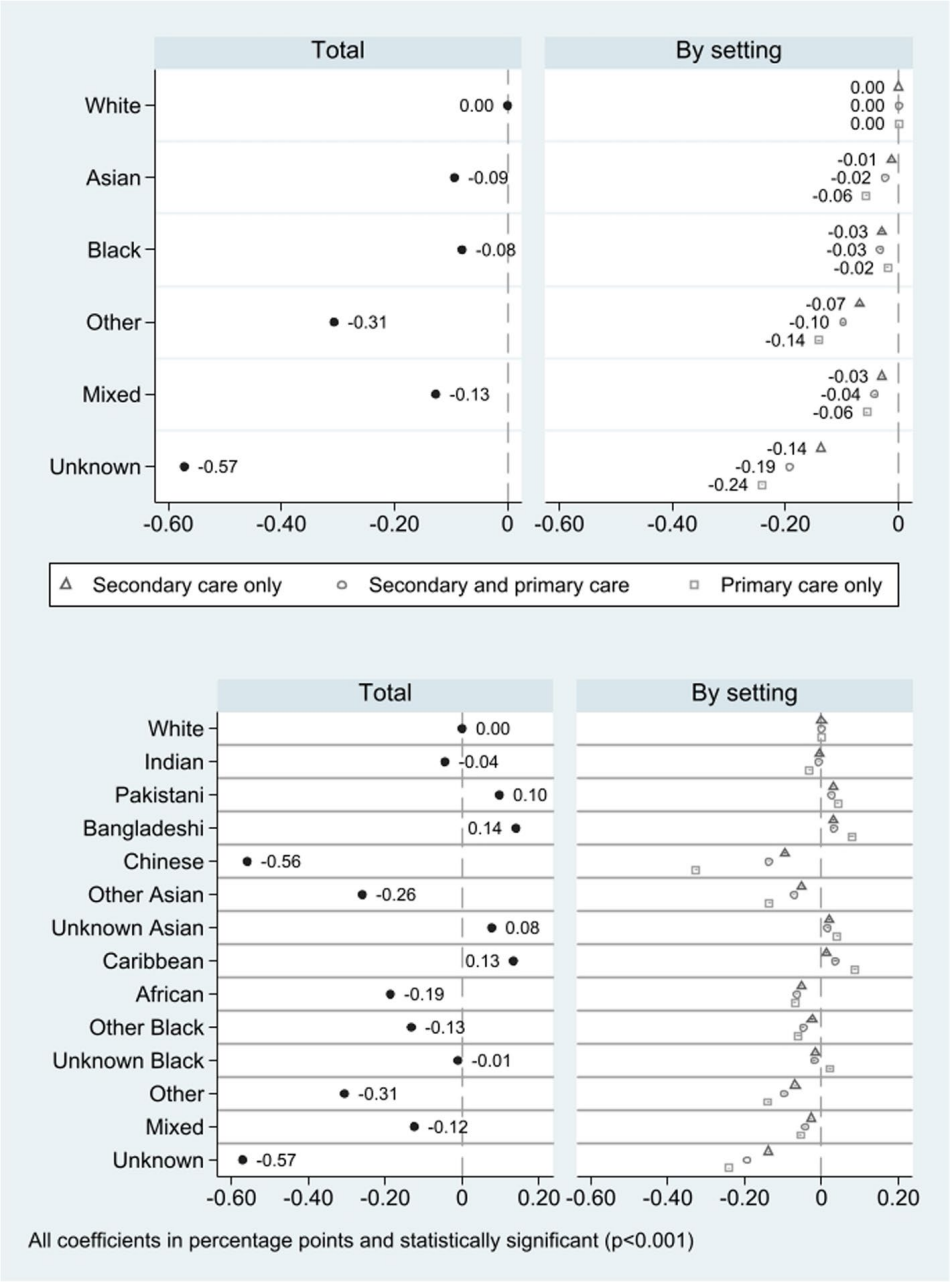
Association between diagnosis recording and ethnicity by setting of recording

Compared to patients of a white ethnic background, patients from all other ethnic groups were significantly less likely to have a diagnosis recorded in any care setting: − 0.08 (95% CI − 0.084 to − 0.076) percentage points (pp) for Black, − 0.09 pp (95% CI − 0.097 to − 0.091) for Asian, − 0.13 pp (95% CI − 0.132 to − 0.122) for mixed, − 0.31 pp (95% CI − 0.312 to − 0.312) for other, and − 0.57 pp (95% CI − 0.575 to 0.570) for unknown ethnic backgrounds (the top panel of Fig. 2; Additional File Table S3).

The difference in the probability of diagnoses recorded in different settings varied across ethnic groups. The differences compared with patients of white ethnic background were larger when considering diagnoses recorded in primary care only, rather than those recorded in both care settings or secondary care only for other (− 0.14 pp, 95% CI − 0.144 to − 0.137 in primary care; − 0.10 pp, 95% CI − 0.100 to − 0.096 in both; − 0.07 pp, 95% CI − 0.071 to − 0.067 in secondary care), mixed (− 0.06 pp, 95% CI − 0.059 to − 0.053; − 0.04 pp, 95% CI − 0.044 to − 0.040; − 0.03 pp, 95% CI 0.031 to − 0.027), and Asian (− 0.06 pp, 95% CI − 0.059 to 0.055; − 0.02 pp, 95% CI − 0.026 to − 0.023; − 0.01 pp, 95% CI − 0.014 to − 0.012) ethnic groups.

The difference in the probability of having a diagnosis recorded between patients of Black and White ethnic backgrounds were similar across settings: − 0.02 pp (95% CI − 0.021 to − 0.017) in primary care only, − 0.03 pp (95% CI − 0.034 to − 0.030) in both settings, and − 0.03 pp (95% CI − 0.031 to − 0.028) for secondary care only.

There were also differences when analysing more refined ethnic groups (the bottom panel of Fig. 2; Additional File Table S3), with the largest discrepancies in diagnostic recording between care settings emerging when comparing patients of Chinese and White ethnic backgrounds. Notably, patients of Chinese ethnic background had a much lower probability of diagnoses recorded in primary care only (− 0.33 percentage points (pp), 95% CI − 0.330 to − 0.323). Compared to patients of a White ethnic group, patients from Pakistani (0.10 pp, 95% CI 0.090 to 0.103), Bangladeshi (0.14 pp, 95% CI 0.130 to 0.150), unknown Asian (0.08 pp, 0.069 to 0.085), and Caribbean (0.13 pp, 0.124 to 0.143) ethnic groups were more likely to have a diagnosis recorded in any setting.

**Fig. 3:**
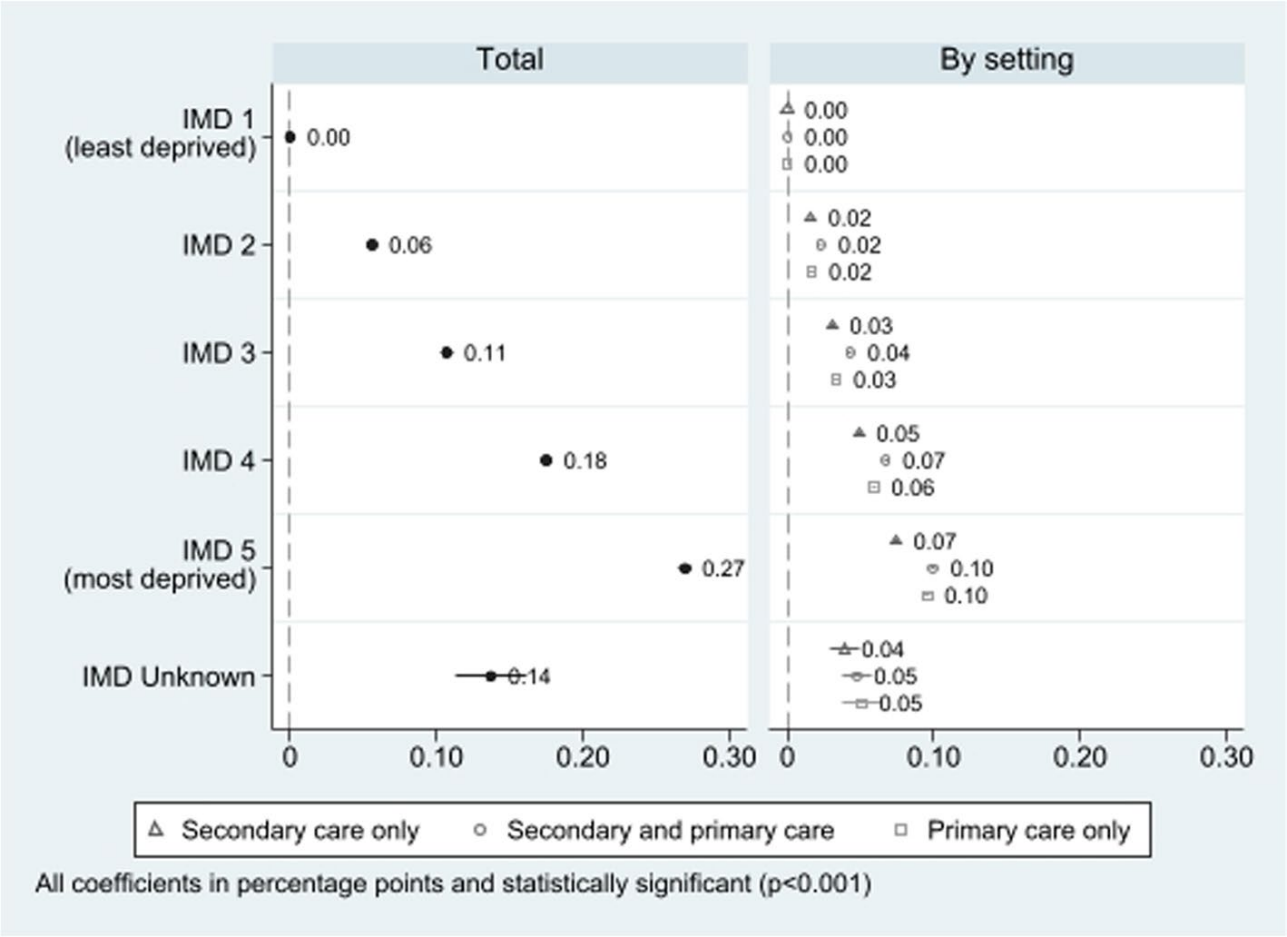
Association between diagnosis recording and deprivation by setting of recording

Patients residing in the most deprived quintile were 0.27 percentage points (pp) (95%IC 0.265 to 0.273) more likely to have a diagnosis recorded in any setting. The gradient was consistent when considering care settings separately, with a slightly less pronounced difference for diagnoses recorded in secondary care only (0.07 pp, 95% CI 0.073 to 0.076) than in primary care only (0.10 pp, 95% CI 0.093 to 0.098) and both settings (0.10 pp, 95% CI 0.098 to 0.101) (Fig. 3; Additional File Table S4)). Patients with unknown area-level deprivation represented only 0.11% of the total.

There was substantially more variation in the probability of diagnosis recording across practices for diagnoses recorded in primary care only compared to those recorded in secondary care only or in both primary and secondary care (Additional File Fig. S1). This is illustrated by the more widespread distribution of practice fixed effects ranging from − 0.411 pp to 0.590 pp (SD 0.0012) for primary care only, compared with a range between − 0.136 pp and 0.268 pp (SD 0.0005) for secondary care only and − 0.212 pp and 0.237 pp (SD 0.0007) for both primary and secondary care.

**Fig. 4:**
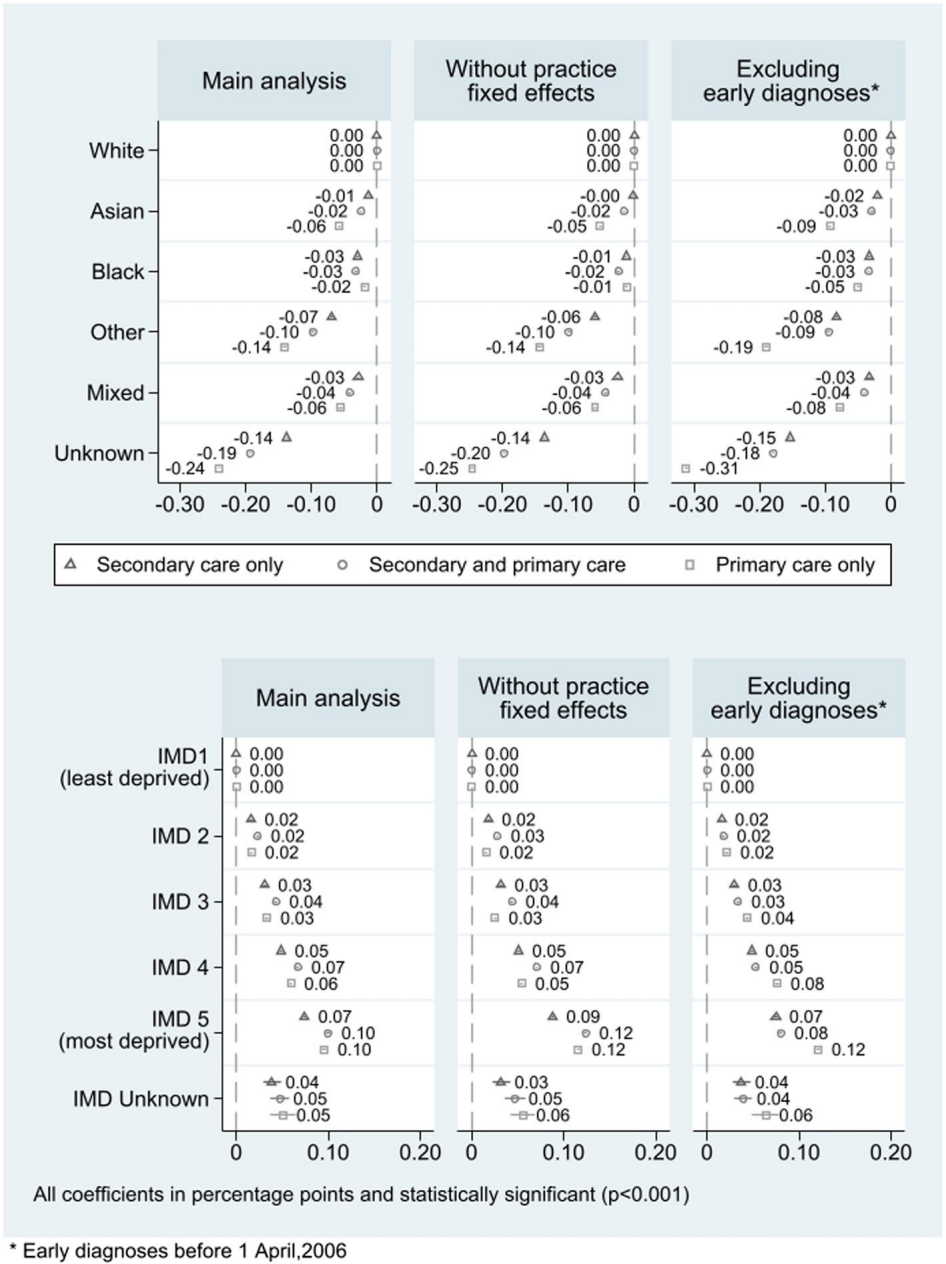
Sensitivity analysis

Inequalities by ethnicity and deprivation were similar when removing the practice fixed effects (Fig. 4; Additional File Table S5). However, patients from a Black ethnic background and patients from the most deprived areas had a higher probability of a diagnosis recorded in any care setting. When we exclusively focused on new diagnoses, the inequalities in diagnostic recording in primary care only, both by ethnic background and by deprivation, were amplified (Fig. 4; Additional File Table S5).

## Discussion

We have shown that there are considerable differences in the recording of chronic diseases between primary and secondary care settings in England, with most diagnoses exclusively recorded in primary care settings. However, a subset of chronic conditions was also diagnosed and recorded exclusively in secondary care settings. Results also suggest small inequalities in the prevalence and recording of chronic conditions by ethnicity, deprivation, and general practice, within 0.6 percentage points (pp). Inequalities are larger in primary care and when using more refined categorisation of ethnic groups. Compared to patients with White ethnic background, Black (− 0.08 pp), Asian (− 0.09 pp), mixed (− 0.13 pp), other (− 0.31 pp), and those of unknown ethnic background (− 0.57 pp) were less likely to have a diagnosis recorded in primary or secondary care. Compared to those residing in less deprived areas, patients residing in the most deprived areas were more likely to have a diagnosis recorded in any setting (0.27 pp), in secondary care only (0.07 pp), in primary care only (0.10 pp), and in both settings (0.10 pp). There was significant variation in the probability of recording across GP practices, particularly when considering diagnoses recorded in primary care only.

This is the first study to investigate inequalities in prevalence captured by diagnostic recording, as well as discrepancies across settings, for a comprehensive set of chronic conditions in England and to identify important differences by ethnicity, deprivation, and providers [1–3]. Differences across ethnic groups are detected and with similar patterns in both primary and secondary care settings. The patterns appear broadly consistent with those of higher life expectancy and lower all-cause mortality [22–24]. However, they cannot be directly compared, for example because our estimates are also adjusted for general practice of registration and in the sensitivity analysis for deprivation.

The category capturing patients with unknown ethnicity (11.56%) has the lowest probability of diagnoses recorded in primary care. Unknown capture patents which had no ethnic code recorded in any setting (7.18%) but also patients that had discordant ethnic groups either between records in CPRD and HES (3.97%) or in CPRD (0.41%). These are also patients with lower healthcare use reflected in a reduced number of records and of younger age. Alternative methods to resolve mismatches in ethnic recording and the use of more complete data sources are being examined, which may, in the future, lead to more complete data [25, 26].

We have built upon previous research on long-term conditions to demonstrate the value of using integrated primary and secondary care datasets to provide a fuller understanding of disease burden of chronic diseases across the English population. We have considered an extended set of 205 long-term conditions for which the prevalence can be consistently mapped and examined in primary or secondary care records. Heterogeneity in multimorbidity prevalence estimates has largely been attributed to the number of conditions included and the mean age of participants [12]. Our results underscore the importance of considering setting alongside inequalities in diagnosis and recording of conditions when selecting chronic conditions for inclusion in studies of multiple long-term conditions [27].

The insights from our study can be used by researchers to examine inequalities in prevalence and trends of specific conditions and of multimorbidity across different populations in England and similar settings [12].

However, there are limitations that need to be considered when interpreting the results. First, we have not considered any information on prescriptions from primary care data to identify chronic diagnoses [1]. However, previous studies reported no alteration in the number of patients with complex health needs when using prescription information alongside administrative medical records [7]. Second, we were unable to analyse to what extent discrepancies and inequalities in diagnostic recording were due to differences in clinical care pathways or disease severity. Individual diagnoses of chronic conditions, such as diabetes or chronic kidney disease, included codes that represent multiple disease stages, and there has been no mapping exercise to subclassify codes between early and late-stage disease. Third, we did not attempt to categorise the chronic conditions analysed according to the setting where they are typically more likely to be managed. However, we excluded 11 conditions typically managed entirely in primary care or outpatient settings. Finally, we could not investigate whether inequalities in recording are associated with specific area level or geographic or primary care provider characteristics because information on general practice is completely anonymised.

Inequalities in diagnostic recording for different patient groups are expected to reflect primarily differences in prevalence. Indeed, differences in recorded prevalence are in line with differences in life expectancy and mortality across groups [22–24]. However, these patterns are also in contrast with studies reporting a higher number of long-term conditions and lower health-related quality of life for some of the categories with lower diagnostic recording [28].

Inequalities could be partially driven by differences in utilisation patterns of primary and secondary care services. 40.77% of diagnoses recorded in secondary care were not recorded in primary care, and 24.40% were only recorded subsequently. 15.26% of the total diagnoses are recorded only in secondary care but not in primary care. This could indicate poor primary care access and quality ahead of hospitalisation and a lack of adequate subsequent follow-up in primary care. Similarly, 62.57% of diagnoses were recorded only in primary care, indicating potentially more effective management of those conditions in primary care. We have controlled for practice fixed effects, which should account for unobserved differences in access and in recording practices. Further research should investigate the reasons for the remaining observed sociodemographic patterns in diagnostic recording and for differences across areas and providers.

Importantly, these discrepancies in diagnostic recording, although small, were patterned by ethnicity, with patients from other, mixed, and Asian ethnic backgrounds having the lowest probabilities of diagnoses reported only in primary care settings. Evidence from some studies suggests that these patients may face persistent barriers to engaging with primary care services [29, 30] or be subject to (implicit) bias in clinicians’ recording practices [31]. Our results highlight the potential implications for diagnosis recording and subsequent care planning of such barriers and bias. Our studies also highlight the importance of more accurately distinguishing ethnic backgrounds, as we demonstrated inequalities within Asian, Black, other, and mixed ethnic groups.

During the period covered by this study, electronic care records in primary care were well developed, but hospitals were often still reliant upon paper notes and patient reporting of diagnoses. This may explain some of the discrepancies in diagnostic reporting noted between primary and secondary care, as clinicians tasked with medical history taking may have not been provided with comprehensive diagnostic information. There is a need for increased investment in integrated electronic health records which can facilitate continuous information sharing of diagnoses between primary and secondary care settings [32].

There is also typically more ownership and involvement of clinicians in diagnostic coding in primary care than in secondary care. GPs routinely review diagnostic coding as they are routinely used for audit and quality improvement purposes, such as for the Quality and Outcomes Framework [33], or the Investment and Impact Fund [34]. However, general practitioners may not code certain conditions, for example plural effusion, and opt instead for the underlying disease, resulting in under-reporting of the condition in primary care compared to secondary care. In contrast, clinical coders in secondary care are responsible for reviewing medical notes to identify relevant diagnostic codes which are specifically related to patient admissions. Therefore, morbidities would be reported only if relevant to the specific episode of care. Further engagement of clinicians working in secondary care settings with improving the consistency and completeness of healthcare administrative datasets, considering their wider use in examining population health, would contribute to maximising their value when used for healthcare quality improvement and planning purposes.

Finally, our study demonstrates the benefits of using linked primary and secondary care datasets for research and healthcare service planning purposes. Estimates of disease burden based on either primary or secondary care datasets in isolation may exclude important diagnoses in a socially patterned way if differences in the recording were not exclusively reflecting prevalence. National-level primary care records exist and can be linked at the person level. Arrangements for utilising them, with appropriate safeguarding and for relevant purposes such as equitable resource allocation, should be considered to advance estimates of disease burden and to understand and tackle inequalities.

## Conclusions

Accurate information on chronic diagnoses is important to inform healthcare service delivery and planning decisions as well as to estimate disease burden across the population. Significant, socially patterned discrepancies in diagnostic recording between primary and secondary care settings emphasise the importance of integrated primary-secondary datasets to map and understand the burden of chronic diseases across the English population. The inequalities detected by ethnicity and deprivation are small but warrant further investigation, with attention to their drivers for specific conditions and multiple long-term conditions. The use of nationally linked primary and secondary care records would enable more accurate estimates of disease burden and detection of inequalities between populations with different ethnic and socio-economic backgrounds, by geography and by primary care providers. The more accurate assessments enabled by linked records is an essential tool to effectively plan resources and services and design policies to tackle inequalities.

## Supplementary Information

Additional file 1: Additional File S1. Procedures for identifying eligible patients from CPRD Aurum. Additional File S2. Algorithm to define ethnicity using linked CPRD Aurum and HES data. Additional File Table S1. Descriptive statistics of socio demographic characteristics for the total population and patients with at least one chronic condition recorded in different settings. Additional File Table S2. Disease prevalence and by settings. Additional File Table S3. Coefficients of ethnic categories estimated from regressions on probability of patients having a diagnosis recorded in different settings. Additional File Table S4. Coefficients of deprivation quintiles estimated from regressions on probability of patients having a diagnosis recorded in different settings. Additional File Fig. S1. Fig. 4. Inequalities in GP practice fixed-effects on diagnostic recording by care setting. Additional File Table S5. Coefficients of ethnic categories estimated from sensitivity analyses removing GP fixed-effects and including more recent diagnoses.

## Data Availability

We used anonymised patient-level longitudinal primary care data from the Clinical Practice Research Datalink (CPRD) Aurum (database version June 2021), linked to the Hospital Episode Statistics (HES) data (set 21) up to October 2020. The protocol was approved by the CPRD Independent Scientific Advisory Committee (ISAC protocol 21–000693). Data can be requested to CPRD.

## Abbreviations

CPRD: Clinical Practice Research Datalink
HES: Hospital Episode Statistics
ICD-10: International Classification of Diseases version 10
IMD: Index of Multiple Deprivation
OPCS: Office of Population, Censuses and Surveys Classification
pp: Percentage points
