# Supplementary material for: Inequalities in the prevalence recording of 205 chronic conditions recorded in primary and secondary care for 12 million patients in the English National Health Service
Source: BMC Med. 2024 Dec 2;22:570. doi: 10.1186/s12916-024-03767-4 (PMC11613489; doi:10.1186/s12916-024-03767-4)
Supplement: Supplementary file 1 — Additional file 1: Additional File S1. Procedures for identifying eligible patients from CPRD Aurum. Additional File S2. Algorithm to define ethnicity using linked CPRD Aurum and HES data. Additional File Table S1. Descriptive statistics of socio demographic characteristics for the total population and patients with at least one chronic condition recorded in different settings. Additional File Table S2. Disease prevalence and by settings. Additional File Table S3. Coefficients of ethnic categories estimated from regressions on probability of patients having a diagnosis recorded in different settings. Additional File Table S4. Coefficients of deprivation quintiles estimated from regressions on probability of patients having a diagnosis recorded in different settings. Additional File Fig. S1. Fig. 4. Inequalities in GP practice fixed-effects on diagnostic recording by care setting. Additional File Table S5. Coefficients of ethnic categories estimated from sensitivity analyses removing GP fixed-effects and including more recent diagnoses. [file 12916_2024_3767_MOESM1_ESM.docx]

**Supplementary Material for Inequalities in the prevalence of 205 chronic conditions recorded in primary and secondary care for 12 million patients in the English National Health Service**

[Additional file S1 – Procedures for identifying eligible patients from CPRD Aurum. 2](#_Toc181881416)

[Additional file S2 – Algorithm to define ethnicity using linked CPRD Aurum and HES data 3](#_Toc181881417)

[Additional file Table S1 – Descriptive statistics of socio demographic characteristics for the total population and patients with at least one chronic condition recorded in different settings. 5](#_Toc181881418)

[Additional file Table S2 – Disease prevalence and by settings. 7](#_Toc181881419)

[Additional file Table S3 – Coefficients of ethnic categories estimated from regressions on probability of patients having a diagnosis recorded in different settings. 15](#_Toc181881420)

[Additional file Table S4 – Coefficients of deprivation quintiles estimated from regressions on probability of patients having a diagnosis recorded in different settings. 16](#_Toc181881421)

[Additional file Figure S1 – Figure 4. Inequalities in GP practice fixed-effects on diagnostic recording by care setting 17](#_Toc181881422)

[Additional file Table S5 – Coefficients of ethnic categories estimated from sensitivity analyses removing GP fixed-effects and including more recent diagnoses 18](#_Toc181881423)

# **Additional file S1 – Procedures for identifying eligible patients from CPRD Aurum.**

We obtained a full sample of patient records from 1,478 GP practices in England from the June 2021 build of the CPRD Aurum.

We excluded 61 GP practices : i) 29 GP practices that appeared likely to have merged into other practices and therefore were recommended for exclusion by the dataset provider (CPRD) to avoid patient duplication; ii) 18 GP practices for which the last data collection happened before the end of our study period – 31st March 2019, hence providing incomplete data; and iii) 14 small practices with list size below 1000 patients.

This leaves 13,135,862 eligible individuals, who were registered with one of the 1,417 GP practices on 1 April 2018 and whose records were flagged by the dataset provider (CPRD) as being of “acceptable” data quality. 12,814,687 (97.3%) of these individuals were registered with a GP practice that has consented to participate in the CPRD and HES data linkage scheme.

In HES, each patient receiving hospital treatment in England from any provider is assigned with a unique patient HESID which allows tracking patients through time and across providers. In CPRD patients are assigned with a patient ID exclusive to each GP practice that the patient has ever registered with, which allows tracking patients through time but not across GP practices.

NHS Digital linked patient IDs in CPRD Aurum with HESID based on the exact match between NHS number and date of birth and/or gender and/or postcode. However, caution should be taken to use the linked data. There were 1,379,938 (10.8%) CPRD patient IDs were linked to the same HES ID attributed to at least one other CPRD ID. We excluded 771 CPRD patient IDs for which the more than 20 CPRD patient IDs were linked to the same HESID or CPRD patient IDs with different age or gender were linked to the same HESID. We further excluded 9,105 patient IDs who were registered with multiple GP practices with overlapping registration periods but have no coinciding medical history records observed.

The final sample includes 12,771,205 (97.2% of the 13,135,862 eligible) patient IDs, of whom 11,832,754 linked to 11,817,417 HESIDs plus 938,451 with no HES data return so not linked to any HESIDs. In total, there are 12,755,868 patients registered with 1,406 GP practices on 1 April, 2018.

# **Additional file S2 – Algorithm to define ethnicity using linked CPRD Aurum and HES data**

We identified ethnicity of patients using records in both primary and secondary care data to increase completeness of available ethnicity information.

The HES linked data include a uniquely defined ethnicity variable for each patient derived from all HES data (including HES outpatient, HES admitted patient care and HES A&E, including 12 mixed level^[[1]](#footnote-1)^ ethnicity classifications: “White”, “Mixed”, “Indian”, “Pakistani”, “Bangladeshi”, “Other Asian”, “Caribbean”, “African”, “Other black”, “Chinese”, “Other” and “Unspecified”. The HES derived variable does not include lower-levels classifications under the higher-level classifications “White” (i.e. “White-British”, “White-Irish” and “White-Any other”) and “Mixed” (i.e. Mixed-White and Black Caribbean”, “Mixed-White and Black African”, “Mixed-White and Asian” and “Mixed-Any other”).

We used the ethnicity codelist by Wright et al (2020) to define the ethnicity in the CPRD Aurum and collapsed them into the 12 classifications to match the HES ethnicity variable.

In general practice, patients can have their ethnicity recorded repeatedly over multiple consultations or visits. Discrepancies may arise if there are mistakes while entering the data or if the patient reports a different ethnic group when asked by the service provider. We combined ethnicity data from the two datasets. If patients had multiple ethnicities recorded in the CPRD, we assume the ethnicity that matched with the HES record is more reliable. If patients had no ethnicity recorded in HES but multiple recorded in CPRD, we following the Mathur et al’s algorithm (2014) (2) to determine the ethnicity depending on whether the ethnicity records was truly matched (identical ethnicity recorded), categorically matched (multiple ethnicities recorded but they fall into the same higher-level classification, i.e. White, Mixed, Asian, Black, Chinese/Other), or truly mismatched (multiple ethnicities spanning different higher-level classifications)^6^.

We further refine the algorithm to adapt to the linked data considering the different ethnicities may be recorded under the same CPRD patient ID or under the different patient IDs that are matched to the same HESID.

Specifically, we used the approach below, and included 14 ethnicity classifications (i.e. “White”, “Indian”, “Pakistani”, “Bangladeshi”, “Other Asian”, “Unknown Asian”, “Chinese”, “Caribbean”, “African”, “Other black”, “Unknown Black”, “Mixed”, “Other” and “Unknown”. This is the same classification used in HES, to which we added “Unknown Asian” and “Unknown African”.

1. For patients with ethnicity recorded in neither CPRD Aurum nor HES (7.18%), we set the ethnicity as “Unknown”.
2. For patients with ethnicity recorded only in HES (37.33%), use the ethnicity in HES.
3. For patients with ethnicity recorded in both CPRD Aurum and HES (45.73%), we:
   1. use the ethnicity in HES if it truly matched to any CPRD record (40.16%).
   2. set the ethnicity as “Unknown category” if it was categorically matched to any CPRD record (1.59%). For example, if a patient was recorded with Indian in HES and Pakistani in CPRD we assign “Unknown Asian”.
   3. set the ethnicity as “Unknown” if it did NOT truly or categorically match to any CPRD record (3.97%).
4. For patients with ethnicity recorded only in CPRD (9.76%),
   1. For patients with one CPRD patient ID linked to one HESID (9.03%), we used the ethnicity in CPRD if only one ethnicity was recorded; and determined the ethnicity depending on whether the records were truly, categorically or not matched, otherwise.
   2. For patients with multiple CPRD patient IDs matched one HESID (0.73%), we used the ethnicity in CPRD if only one has ever been recorded; if only one of the patient ID had multiple ethnicities recorded, we determined the ethnicity by checking the matching ethnicity across CPRD patient IDs; if more than one patient IDs had multiple ethnicities recorded, we set the ethnicity as “Unknown”.

# **Additional file Table S1 – Descriptive statistics of socio demographic characteristics for the total population and patients with at least one chronic condition recorded in different settings.**

|  | **Population** | |  | **Patients with at least one chronic condition recorded in** | | | | | | | | |
| --- | --- | --- | --- | --- | --- | --- | --- | --- | --- | --- | --- | --- |
|  |  |  |  | **secondary care only** | | **secondary and primary care** | | **primary care only** | |  | **secondary or primary care** | |
|  | **N** | **(%)** |  | **N** | **(%)** | **N** | **(%)** | **N** | **(%)** |  | **N** | **(%)** |
| Number of patients | 12,755,868 |  |  | 2,586,399 |  | 3,341,743 |  | 8,671,633 |  |  | 9,267,558 |  |
|  |  |  |  |  |  |  |  |  |  |  |  |  |
| Age, years | |  |  |  |  |  |  |  |  |  |  |  |
| <10 year | 1,408,291 | (11.04%) |  | 65,021 | (2.51%) | 82,651 | (2.47%) | 525,853 | (6.06%) |  | 597,552 | (6.45%) |
| 11-19 years | 1,429,010 | (11.20%) |  | 85,375 | (3.30%) | 117,672 | (3.52%) | 760,949 | (8.78%) |  | 824,595 | (8.90%) |
| 21-29 years | 1,801,690 | (14.12%) |  | 163,068 | (6.30%) | 192,942 | (5.77%) | 1,105,314 | (12.75%) |  | 1,167,928 | (12.60%) |
| 31-39 years | 1,891,504 | (14.83%) |  | 252,570 | (9.77%) | 310,621 | (9.30%) | 1,199,634 | (13.83%) |  | 1,294,289 | (13.97%) |
| 41-49 years | 1,702,256 | (13.34%) |  | 291,900 | (11.29%) | 379,745 | (11.36%) | 1,217,556 | (14.04%) |  | 1,301,294 | (14.04%) |
| 51-59 years | 1,693,277 | (13.27%) |  | 414,526 | (16.03%) | 552,150 | (16.52%) | 1,336,060 | (15.41%) |  | 1,423,131 | (15.36%) |
| 61-69 years | 1,247,262 | (9.78%) |  | 430,907 | (16.66%) | 587,932 | (17.59%) | 1,063,389 | (12.26%) |  | 1,128,846 | (12.18%) |
| 71-79 years | 965,800 | (7.57%) |  | 469,340 | (18.15%) | 620,268 | (18.56%) | 878,385 | (10.13%) |  | 924,090 | (9.97%) |
| 80 years or older | 616,778 | (4.84%) |  | 413,692 | (15.99%) | 497,762 | (14.90%) | 584,493 | (6.74%) |  | 605,833 | (6.54%) |
|  |  |  |  |  |  |  |  |  |  |  |  |  |
| Gender |  |  |  |  |  |  |  |  |  |  |  |  |
| Female | 6,361,122 | (49.87%) |  | 1,396,374 | (53.99%) | 1,810,872 | (54.19%) | 4,565,712 | (52.65%) |  | 4,847,800 | (52.31%) |
| Male | 6,394,746 | (50.13%) |  | 1,190,025 | (46.01%) | 1,530,871 | (45.81%) | 4,105,921 | (47.35%) |  | 4,419,758 | (47.69%) |
|  |  |  |  |  |  |  |  |  |  |  |  |  |
| Ethnicity |  |  |  |  |  |  |  |  |  |  |  |  |
| White | 9,358,814 | (73.37%) |  | 2,218,651 | (85.78%) | 2,873,331 | (85.98%) | 6,709,206 | (77.37%) |  | 7,191,855 | (77.60%) |
| Asian | 1,041,689 | (8.17%) |  | 143,058 | (5.53%) | 183,530 | (5.49%) | 640,327 | (7.38%) |  | 677,825 | (7.31%) |
| -Indian | 307,064 | (2.41%) |  | 48,156 | (1.86%) | 61,940 | (1.85%) | 48,156 | (1.86%) |  | 208,289 | (2.25%) |
| -Pakistani | 224,769 | (1.76%) |  | 34,971 | (1.35%) | 45,345 | (1.36%) | 34,971 | (1.35%) |  | 157,400 | (1.70%) |
| -Bangladeshi | 95,948 | (0.75%) |  | 13,085 | (0.51%) | 16,952 | (0.51%) | 13,085 | (0.51%) |  | 69,509 | (0.75%) |
| -Chinese | 114,252 | (0.90%) |  | 6,965 | (0.27%) | 8,595 | (0.26%) | 6,965 | (0.27%) |  | 46,405 | (0.50%) |
| -Other Asian | 177,180 | (1.39%) |  | 19,140 | (0.74%) | 24,091 | (0.72%) | 19,140 | (0.74%) |  | 105,105 | (1.13%) |
| -Unknown Asian | 122,476 | (0.96%) |  | 20,741 | (0.80%) | 26,607 | (0.80%) | 20,741 | (0.80%) |  | 91,117 | (0.98%) |
| Black | 543,020 | (4.26%) |  | 86,124 | (3.33%) | 111,521 | (3.34%) | 362,277 | (4.18%) |  | 386,861 | (4.17%) |
| -Caribbean | 121,437 | (0.95%) |  | 29,640 | (1.15%) | 38,257 | (1.14%) | 29,640 | (1.15%) |  | 98,132 | (1.06%) |
| -African | 278,720 | (2.19%) |  | 34,202 | (1.32%) | 44,852 | (1.34%) | 34,202 | (1.32%) |  | 185,075 | (2.00%) |
| -Other Black | 44,067 | (0.35%) |  | 5,579 | (0.22%) | 6,624 | (0.20%) | 5,579 | (0.22%) |  | 28,860 | (0.31%) |
| -Unknown Black | 98,796 | (0.77%) |  | 16,703 | (0.65%) | 21,788 | (0.65%) | 16,703 | (0.65%) |  | 74,794 | (0.81%) |
| Other | 169,428 | (1.33%) |  | 17,910 | (0.69%) | 20,618 | (0.62%) | 89,522 | (1.03%) |  | 96,025 | (1.04%) |
| Mixed | 168,832 | (1.32%) |  | 16,022 | (0.62%) | 20,082 | (0.60%) | 90,816 | (1.05%) |  | 98,362 | (1.06%) |
| Unknown | 1,474,085 | (11.56%) |  | 104,634 | (4.05%) | 132,661 | (3.97%) | 779,485 | (8.99%) |  | 816,630 | (8.81%) |
|  |  |  |  |  |  |  |  |  |  |  |  |  |
| IMD quintile | |  |  |  |  |  |  |  |  |  |  |  |
| IMD1 (least deprived) | 2,556,134 | (20.04%) |  | 506,946 | (19.60%) | 668,531 | (20.01%) | 1,785,106 | (20.59%) |  | 1,900,154 | (20.50%) |
| IMD2 | 2,540,909 | (19.92%) |  | 522,633 | (20.21%) | 684,657 | (20.49%) | 1,755,922 | (20.25%) |  | 1,873,889 | (20.22%) |
| IMD3 | 2,489,372 | (19.52%) |  | 501,969 | (19.41%) | 646,683 | (19.35%) | 1,679,050 | (19.36%) |  | 1,794,721 | (19.37%) |
| IMD4 | 2,675,092 | (20.97%) |  | 522,681 | (20.21%) | 665,571 | (19.92%) | 1,770,151 | (20.41%) |  | 1,893,088 | (20.43%) |
| IMD5 (most deprived) | 2,480,517 | (19.45%) |  | 529,488 | (20.47%) | 672,818 | (20.13%) | 1,672,038 | (19.28%) |  | 1,795,667 | (19.38%) |
| IMD unknown | 13,844 | (0.11%) |  | 2,682 | (0.10%) | 3,483 | (0.10%) | 9,366 | (0.11%) |  | 10,039 | (0.11%) |
|  |  |  |  |  |  |  |  |  |  |  |  |  |
| Newly registered patient | 1,164,262 | (9.13%) |  | 156,708 | (6.06%) | 184,566 | (5.52%) | 621,574 | (7.17%) |  | 671,756 | (7.25%) |

# **Additional file Table S2 – Disease prevalence and by settings.**

| **Disease** | **Prevalence among the full population recorded in** | | | |  | **% Diagnosed cases recorded in** | | |
| --- | --- | --- | --- | --- | --- | --- | --- | --- |
|  | **In total** | **secondary care only** | **secondary and**  **primary care** | **primary care**  **only** |  | **secondary care**  **only** | **secondary and**  **primary care** | **primary care**  **only** |
| **Cancers** |  |  |  |  |  |  |  |  |
| All cancers | 4.94% | 0.58% | 2.98% | 1.38% |  | 11.78% | 60.30% | 27.91% |
| Primary malignancy_skin | 1.83% | 0.23% | 0.77% | 0.84% |  | 12.49% | 41.89% | 45.62% |
| Primary malignancy_breast | 0.85% | 0.05% | 0.64% | 0.16% |  | 5.48% | 75.42% | 19.10% |
| Primary malignancy_prostate | 0.61% | 0.06% | 0.39% | 0.16% |  | 9.75% | 63.80% | 26.44% |
| Primary malignancy_bowel | 0.37% | 0.06% | 0.26% | 0.05% |  | 15.94% | 70.63% | 13.43% |
| Secondary malignancy_lymph nodes | 0.33% | 0.32% | 0.01% | 0.00% |  | 96.24% | 2.43% | 1.33% |
| Primary malignancy_melanoma | 0.30% | 0.02% | 0.12% | 0.16% |  | 6.60% | 40.05% | 53.34% |
| Primary malignancy_other | 0.23% | 0.13% | 0.04% | 0.06% |  | 57.05% | 17.51% | 25.44% |
| Primary malignancy_bladder | 0.22% | 0.07% | 0.13% | 0.02% |  | 32.83% | 57.96% | 9.22% |
| Non-Hodgkin lymphoma | 0.18% | 0.03% | 0.11% | 0.04% |  | 16.11% | 61.95% | 21.93% |
| Leukaemia | 0.13% | 0.02% | 0.07% | 0.04% |  | 14.88% | 56.50% | 28.62% |
| Secondary malignancy_other | 0.13% | 0.04% | 0.01% | 0.07% |  | 34.88% | 11.17% | 53.95% |
| Primary malignancy_lung | 0.12% | 0.03% | 0.08% | 0.02% |  | 22.12% | 63.79% | 14.08% |
| Primary malignancy_kidney | 0.11% | 0.04% | 0.05% | 0.02% |  | 40.25% | 45.21% | 14.52% |
| Primary malignancy_uterus | 0.10% | 0.03% | 0.06% | 0.01% |  | 29.94% | 58.06% | 12.00% |
| Primary malignancy_oropharyngeal | 0.09% | 0.04% | 0.04% | 0.01% |  | 39.96% | 44.49% | 15.55% |
| Secondary malignancy_bone | 0.08% | 0.07% | 0.01% | 0.00% |  | 86.49% | 9.08% | 4.43% |
| Primary malignancy_ovary | 0.08% | 0.02% | 0.03% | 0.03% |  | 24.54% | 38.78% | 36.67% |
| Polycythaemia vera | 0.07% | 0.02% | 0.02% | 0.03% |  | 25.56% | 29.93% | 44.49% |
| Secondary malignancy_liver | 0.06% | 0.04% | 0.02% | 0.01% |  | 59.71% | 32.28% | 8.00% |
| Secondary malignancy_lung | 0.06% | 0.05% | 0.01% | 0.00% |  | 86.95% | 8.97% | 4.06% |
| Multiple myeloma and malignant plasma  cell neoplasms | 0.06% | 0.01% | 0.04% | 0.01% |  | 18.75% | 66.95% | 14.30% |
| Primary malignancy_thyroid | 0.05% | 0.01% | 0.03% | 0.01% |  | 24.27% | 51.39% | 24.34% |
| Primary malignancy_testis | 0.05% | 0.01% | 0.03% | 0.01% |  | 16.33% | 54.57% | 29.12% |
| Primary malignancy_brain | 0.04% | 0.01% | 0.02% | 0.01% |  | 30.68% | 44.09% | 25.23% |
| Primary malignancy_cervical | 0.04% | 0.01% | 0.02% | 0.01% |  | 21.41% | 50.42% | 28.17% |
| Hodgkin lymphoma | 0.04% | 0.01% | 0.02% | 0.01% |  | 20.83% | 56.23% | 22.94% |
| Myelodysplastic syndromes | 0.04% | 0.01% | 0.01% | 0.01% |  | 31.89% | 39.46% | 28.64% |
| Primary malignancy_oesophageal | 0.04% | 0.01% | 0.02% | 0.00% |  | 20.01% | 67.36% | 12.66% |
| Secondary malignancy_peritoneum | 0.03% | 0.03% | 0.00% | 0.00% |  | 96.54% | 2.20% | 1.28% |
| Primary malignancy_stomach | 0.03% | 0.02% | 0.01% | 0.00% |  | 50.68% | 37.94% | 11.38% |
| Primary malignancy_multiple sites | 0.03% | 0.03% | 0.00% | 0.00% |  | 99.64% | 0.03% | 0.34% |
| Primary malignancy_pancreas | 0.02% | 0.01% | 0.01% | 0.00% |  | 39.77% | 42.48% | 17.75% |
| Primary malignancy_bone | 0.02% | 0.01% | 0.01% | 0.01% |  | 35.95% | 30.70% | 33.35% |
| Primary malignancy_liver | 0.01% | 0.00% | 0.01% | 0.00% |  | 28.97% | 50.83% | 20.14% |
| Secondary malignancy_brain | 0.01% | 0.01% | 0.00% | 0.00% |  | 57.85% | 33.00% | 9.15% |
| Secondary malignancy_pleura | 0.01% | 0.01% | 0.00% | 0.00% |  | 98.98% | 0.67% | 0.40% |
| Primary malignancy_biliary tract | 0.01% | 0.00% | 0.00% | 0.00% |  | 41.27% | 40.23% | 18.40% |
| Secondary malignancy_bowel | 0.01% | 0.01% | 0.00% | 0.00% |  | 96.76% | 0.66% | 2.65% |
| Secondary malignancy_adrenal gland | 0.01% | 0.01% | 0.00% | 0.00% |  | 94.84% | 2.69% | 2.45% |
| Primary malignancy_mesothelioma | 0.00% | 0.00% | 0.00% | 0.00% |  | 70.84% | 25.49% | 3.39% |
|  |  |  |  |  |  |  |  |  |
| **Diseases of the Circulatory System** |  |  |  |  |  |  |  |  |
| Hypertension | 15.46% | 2.46% | 7.52% | 5.48% |  | 15.93% | 48.61% | 35.46% |
| Coronary heart disease NOS | 3.38% | 1.91% | 0.90% | 0.57% |  | 56.51% | 26.51% | 16.99% |
| Atrial fibrillation | 2.54% | 0.52% | 1.52% | 0.49% |  | 20.66% | 60.11% | 19.23% |
| Stable angina | 2.31% | 0.87% | 0.81% | 0.63% |  | 37.53% | 35.23% | 27.24% |
| Myocardial infarction | 1.63% | 0.70% | 0.83% | 0.10% |  | 43.15% | 50.68% | 6.17% |
| Heart failure | 1.49% | 0.51% | 0.54% | 0.44% |  | 34.44% | 36.04% | 29.52% |
| Stroke NOS | 1.12% | 0.12% | 0.15% | 0.85% |  | 10.32% | 13.83% | 75.85% |
| Transient ischaemic attack | 0.96% | 0.20% | 0.22% | 0.54% |  | 21.08% | 23.06% | 55.86% |
| Venous thromboembolic disease (Excl PE) | 0.79% | 0.09% | 0.25% | 0.45% |  | 11.89% | 31.08% | 57.02% |
| Peripheral arterial disease | 0.79% | 0.20% | 0.27% | 0.32% |  | 24.76% | 34.37% | 40.88% |
| Raynaud's syndrome | 0.70% | 0.07% | 0.07% | 0.55% |  | 10.31% | 10.59% | 79.10% |
| Nonrheumatic aortic valve disorders | 0.67% | 0.19% | 0.23% | 0.25% |  | 28.39% | 34.75% | 36.86% |
| Ischaemic stroke | 0.65% | 0.38% | 0.16% | 0.11% |  | 59.20% | 24.25% | 16.55% |
| Nonrheumatic mitral valve disorders | 0.59% | 0.22% | 0.11% | 0.26% |  | 37.02% | 19.12% | 43.86% |
| Unstable angina | 0.50% | 0.34% | 0.06% | 0.09% |  | 69.09% | 12.79% | 18.13% |
| Right bundle branch block combinations | 0.48% | 0.31% | 0.05% | 0.12% |  | 63.79% | 10.26% | 25.96% |
| Atrioventricular blocks | 0.44% | 0.35% | 0.03% | 0.06% |  | 79.16% | 7.42% | 13.42% |
| Supraventricular tachycardia | 0.41% | 0.15% | 0.12% | 0.14% |  | 36.34% | 28.59% | 35.08% |
| Multiple valve disorder | 0.41% | 0.37% | 0.01% | 0.03% |  | 90.39% | 2.90% | 6.71% |
| Left bundle branch block | 0.37% | 0.23% | 0.07% | 0.07% |  | 62.83% | 19.03% | 18.15% |
| Abdominal aortic aneurysm | 0.23% | 0.12% | 0.06% | 0.05% |  | 51.54% | 26.30% | 22.16% |
| Other cardiomyopathy | 0.18% | 0.07% | 0.04% | 0.07% |  | 39.58% | 23.20% | 37.22% |
| Rheumatic valve disorder | 0.13% | 0.11% | 0.01% | 0.02% |  | 80.00% | 7.00% | 13.00% |
| Intracerebral haemorrhage | 0.13% | 0.05% | 0.04% | 0.03% |  | 39.94% | 34.16% | 25.90% |
| Pericardial effusion | 0.12% | 0.08% | 0.02% | 0.02% |  | 69.35% | 16.75% | 13.90% |
| Ventricular tachycardia | 0.11% | 0.07% | 0.02% | 0.02% |  | 60.92% | 21.25% | 17.83% |
| Secondary pulmonary hypertension | 0.11% | 0.08% | 0.01% | 0.01% |  | 76.32% | 11.03% | 12.66% |
| Subarachnoid haemorrhage | 0.11% | 0.02% | 0.05% | 0.04% |  | 19.29% | 43.45% | 37.28% |
| Dilated cardiomyopathy | 0.11% | 0.06% | 0.03% | 0.02% |  | 55.87% | 27.89% | 16.22% |
| Primary pulmonary hypertension | 0.08% | 0.04% | 0.01% | 0.03% |  | 50.38% | 12.14% | 37.48% |
| Sick sinus syndrome | 0.07% | 0.05% | 0.01% | 0.01% |  | 73.11% | 13.75% | 13.14% |
| Hypertrophic cardiomyopathy | 0.05% | 0.01% | 0.02% | 0.02% |  | 26.72% | 39.95% | 33.33% |
|  |  |  |  |  |  |  |  |  |
| **Diseases of the Digestive System** |  |  |  |  |  |  |  |  |
| Gastro-oesophageal reflux disease | 7.46% | 1.32% | 0.94% | 5.20% |  | 17.66% | 12.64% | 69.70% |
| Gastritis and duodenitis | 4.37% | 1.45% | 1.39% | 1.53% |  | 33.24% | 31.78% | 34.98% |
| Abdominal hernia | 3.83% | 0.38% | 1.88% | 1.57% |  | 9.90% | 49.00% | 41.09% |
| Irritable bowel syndrome | 3.52% | 0.44% | 0.36% | 2.72% |  | 12.43% | 10.30% | 77.27% |
| Diverticular disease of intestine | 3.33% | 1.03% | 1.69% | 0.61% |  | 30.88% | 50.75% | 18.37% |
| Diaphragmatic hernia | 3.20% | 1.22% | 1.53% | 0.45% |  | 38.17% | 47.93% | 13.90% |
| Fatty Liver | 0.86% | 0.29% | 0.08% | 0.50% |  | 33.12% | 8.87% | 58.02% |
| Ulcerative colitis | 0.49% | 0.14% | 0.26% | 0.09% |  | 27.80% | 53.72% | 18.48% |
| Barrett's oesophagus | 0.42% | 0.07% | 0.26% | 0.09% |  | 17.09% | 61.82% | 21.09% |
| Pancreatitis | 0.37% | 0.09% | 0.22% | 0.06% |  | 24.24% | 59.47% | 16.29% |
| Crohn's disease | 0.34% | 0.09% | 0.20% | 0.05% |  | 25.58% | 58.64% | 15.77% |
| Coeliac disease | 0.32% | 0.05% | 0.15% | 0.13% |  | 15.00% | 45.91% | 39.09% |
| Cirrhosis | 0.23% | 0.07% | 0.09% | 0.07% |  | 32.60% | 38.82% | 28.57% |
| Alcoholic liver disease | 0.19% | 0.05% | 0.04% | 0.10% |  | 25.59% | 22.30% | 52.11% |
| Portal hypertension | 0.09% | 0.06% | 0.02% | 0.01% |  | 69.03% | 23.92% | 7.07% |
| Oesophageal varices | 0.08% | 0.04% | 0.04% | 0.01% |  | 44.13% | 49.41% | 6.46% |
| Autoimmune liver disease | 0.06% | 0.01% | 0.03% | 0.03% |  | 14.82% | 42.03% | 43.16% |
| Hepatic failure | 0.06% | 0.05% | 0.01% | 0.01% |  | 76.57% | 15.35% | 8.09% |
| Angiodysplasia of colon | 0.06% | 0.04% | 0.01% | 0.00% |  | 72.71% | 18.93% | 8.38% |
|  |  |  |  |  |  |  |  |  |
| **Diseases of the Ear** |  |  |  |  |  |  |  |  |
| Hearing loss | 5.51% | 0.43% | 0.47% | 4.61% |  | 7.74% | 8.51% | 83.75% |
| Tinnitus | 1.99% | 0.05% | 0.03% | 1.91% |  | 2.34% | 1.47% | 96.19% |
| Meniere disease | 0.18% | 0.03% | 0.04% | 0.12% |  | 17.60% | 19.64% | 62.77% |
|  |  |  |  |  |  |  |  |  |
| **Diseases of the Endocrine System** |  |  |  |  |  |  |  |  |
| Obesity | 20.99% | 0.46% | 2.85% | 17.68% |  | 2.19% | 13.59% | 84.22% |
| Diabetes | 6.01% | 0.26% | 3.29% | 2.45% |  | 4.37% | 54.82% | 40.82% |
| Thyroid disease | 3.97% | 0.46% | 1.62% | 1.89% |  | 11.59% | 40.85% | 47.56% |
| Polycystic ovarian syndrome | 0.62% | 0.22% | 0.09% | 0.32% |  | 35.42% | 13.75% | 50.83% |
| Hyperparathyroidism | 0.19% | 0.04% | 0.07% | 0.09% |  | 19.64% | 33.57% | 46.79% |
| Cystic fibrosis | 0.02% | 0.00% | 0.01% | 0.00% |  | 22.03% | 57.27% | 20.70% |
|  |  |  |  |  |  |  |  |  |
| **Diseases of the Eye** |  |  |  |  |  |  |  |  |
| Cataract | 4.29% | 1.03% | 2.22% | 1.05% |  | 23.91% | 51.72% | 24.38% |
| Diabetic eye disease | 2.14% | 0.08% | 0.28% | 1.78% |  | 3.94% | 13.13% | 82.93% |
| Glaucoma | 1.22% | 0.21% | 0.42% | 0.60% |  | 17.28% | 34.03% | 48.69% |
| Macular degeneration | 0.98% | 0.31% | 0.21% | 0.46% |  | 31.13% | 21.86% | 47.01% |
| Visual impairment and blindness | 0.76% | 0.16% | 0.10% | 0.50% |  | 21.17% | 12.91% | 65.92% |
| Anterior and intermediate uveitis | 0.41% | 0.02% | 0.03% | 0.36% |  | 5.31% | 7.45% | 87.24% |
| Retinal vascular occlusions | 0.27% | 0.03% | 0.05% | 0.19% |  | 10.00% | 19.94% | 70.07% |
| Scleritis and episcleritis | 0.25% | 0.00% | 0.00% | 0.24% |  | 1.04% | 1.38% | 97.58% |
| Posterior uveitis | 0.03% | 0.00% | 0.00% | 0.02% |  | 9.51% | 6.62% | 83.87% |
|  |  |  |  |  |  |  |  |  |
| **Diseases of the Genitourinary System** |  |  |  |  |  |  |  |  |
| Chronic kidney disease | 6.02% | 0.04% | 0.86% | 5.12% |  | 0.66% | 14.31% | 85.04% |
| Urinary incontinence | 2.51% | 0.17% | 0.26% | 2.08% |  | 6.73% | 10.18% | 83.10% |
| Hyperplasia of prostate | 2.00% | 0.62% | 0.43% | 0.96% |  | 30.96% | 21.24% | 47.80% |
| Dysmenorrhoea | 1.56% | 0.12% | 0.05% | 1.39% |  | 7.59% | 2.97% | 89.45% |
| Neuropahtic bladder | 0.85% | 0.06% | 0.04% | 0.75% |  | 7.31% | 4.31% | 88.37% |
| Endometriosis | 0.72% | 0.25% | 0.22% | 0.25% |  | 35.10% | 30.95% | 33.95% |
| Obstructive and reflux uropathy | 0.56% | 0.32% | 0.11% | 0.13% |  | 56.91% | 19.45% | 23.63% |
| Tubulo-interstitial nephritis | 0.32% | 0.31% | 0.00% | 0.00% |  | 98.18% | 0.69% | 1.13% |
| Glomerulonephritis | 0.28% | 0.18% | 0.05% | 0.05% |  | 62.80% | 19.22% | 17.99% |
| Endometrial hyperplasia and hypertrophy | 0.23% | 0.18% | 0.01% | 0.04% |  | 78.21% | 4.54% | 17.26% |
| Chronic cystitis | 0.13% | 0.07% | 0.02% | 0.04% |  | 54.90% | 13.61% | 31.49% |
|  |  |  |  |  |  |  |  |  |
| **Diseases of the Respiratory System** |  |  |  |  |  |  |  |  |
| Asthma | 10.75% | 1.98% | 3.76% | 5.00% |  | 18.43% | 35.02% | 46.56% |
| Allergic and chronic rhinitis | 9.03% | 0.24% | 0.20% | 8.59% |  | 2.66% | 2.24% | 95.10% |
| COPD | 2.41% | 0.55% | 1.00% | 0.87% |  | 22.68% | 41.36% | 35.96% |
| Chronic sinusitis | 1.46% | 0.18% | 0.09% | 1.19% |  | 12.44% | 5.84% | 81.71% |
| Sleep apnoea | 1.11% | 0.33% | 0.35% | 0.42% |  | 29.98% | 32.03% | 37.99% |
| Pleural effusion | 0.80% | 0.64% | 0.11% | 0.06% |  | 79.29% | 13.48% | 7.23% |
| Bronchiectasis | 0.44% | 0.09% | 0.16% | 0.18% |  | 20.88% | 36.95% | 42.17% |
| Hypertrophic nasal turbinates | 0.26% | 0.21% | 0.01% | 0.04% |  | 79.91% | 3.50% | 16.59% |
| Pleural plaque | 0.14% | 0.07% | 0.02% | 0.05% |  | 47.04% | 16.41% | 36.55% |
| Pulmonary fibrosis | 0.14% | 0.06% | 0.04% | 0.04% |  | 44.77% | 26.02% | 29.21% |
| Asbestosis | 0.07% | 0.01% | 0.01% | 0.04% |  | 21.71% | 15.31% | 62.98% |
|  |  |  |  |  |  |  |  |  |
| **Haematological/Immunological conditions** | | |  |  |  |  |  |  |
| Other anaemias | 3.44% | 1.30% | 0.50% | 1.64% |  | 37.79% | 14.64% | 47.58% |
| Vitamin B12 deficiency anaemia | 1.67% | 0.02% | 0.10% | 1.55% |  | 1.49% | 5.88% | 92.62% |
| Folate deficiency anaemia | 0.55% | 0.05% | 0.00% | 0.50% |  | 8.63% | 0.78% | 90.59% |
| Secondary thrombocytopaenia | 0.35% | 0.15% | 0.05% | 0.15% |  | 43.13% | 14.20% | 42.66% |
| Thrombophilia | 0.22% | 0.02% | 0.03% | 0.17% |  | 10.80% | 12.60% | 76.60% |
| Hypersplenism | 0.13% | 0.07% | 0.02% | 0.04% |  | 56.39% | 12.33% | 31.27% |
| Sarcoidosis | 0.12% | 0.02% | 0.05% | 0.05% |  | 16.27% | 38.71% | 45.02% |
| Primary thrombocytopaenia | 0.09% | 0.03% | 0.03% | 0.03% |  | 34.16% | 34.37% | 31.48% |
| Hyposplenism | 0.09% | 0.03% | 0.03% | 0.03% |  | 31.68% | 30.86% | 37.46% |
| Thalassaemia | 0.08% | 0.04% | 0.01% | 0.04% |  | 45.41% | 9.43% | 45.16% |
| Secondary polycythaemia | 0.08% | 0.03% | 0.01% | 0.03% |  | 41.52% | 17.40% | 41.08% |
| Other haemolytic anaemias | 0.07% | 0.03% | 0.02% | 0.03% |  | 34.36% | 25.57% | 40.06% |
| Aplastic anaemias | 0.07% | 0.04% | 0.02% | 0.02% |  | 54.08% | 23.12% | 22.80% |
| Immunodeficiencies | 0.05% | 0.02% | 0.01% | 0.01% |  | 49.41% | 22.22% | 28.35% |
| Sickle cell anaemia | 0.05% | 0.02% | 0.02% | 0.01% |  | 41.83% | 44.02% | 14.13% |
|  |  |  |  |  |  |  |  |  |
| **Infectious Diseases** |  |  |  |  |  |  |  |  |
| Chronic viral hepatitis | 0.28% | 0.05% | 0.08% | 0.16% |  | 17.43% | 27.18% | 55.38% |
| HIV | 0.13% | 0.00% | 0.01% | 0.11% |  | 3.65% | 8.88% | 87.47% |
|  |  |  |  |  |  |  |  |  |
| Mental Health Disorders |  |  |  |  |  |  |  |  |
| Depression | 11.63% | 1.14% | 2.01% | 8.48% |  | 9.79% | 17.28% | 72.93% |
| Anxiety disorders | 11.36% | 1.05% | 1.24% | 9.07% |  | 9.23% | 10.90% | 79.87% |
| Alcohol misuse | 4.49% | 0.45% | 0.50% | 3.54% |  | 10.00% | 11.22% | 78.78% |
| Substance misuse | 1.36% | 0.27% | 0.24% | 0.85% |  | 20.18% | 17.58% | 62.24% |
| Dementia | 0.97% | 0.11% | 0.43% | 0.43% |  | 11.74% | 44.16% | 44.10% |
| Intellectual disability | 0.70% | 0.15% | 0.18% | 0.37% |  | 21.80% | 25.81% | 52.39% |
| Autism and Asperger's syndrome | 0.62% | 0.07% | 0.13% | 0.43% |  | 10.68% | 20.41% | 68.92% |
| Schizophrenia | 0.60% | 0.12% | 0.24% | 0.25% |  | 20.42% | 38.93% | 40.65% |
| Hyperkinetic disorders | 0.41% | 0.04% | 0.07% | 0.31% |  | 9.24% | 16.12% | 74.64% |
| Bipolar affective disorder and mania | 0.37% | 0.10% | 0.14% | 0.13% |  | 26.24% | 38.20% | 35.57% |
| Personality disorders | 0.37% | 0.06% | 0.09% | 0.21% |  | 17.17% | 25.50% | 57.33% |
| Obsessive-compulsive disorder | 0.31% | 0.03% | 0.03% | 0.25% |  | 9.22% | 10.14% | 80.63% |
| Anorexia and bulimia nervosa | 0.17% | 0.02% | 0.03% | 0.12% |  | 11.46% | 17.11% | 71.43% |
|  |  |  |  |  |  |  |  |  |
| **Musculoskeletal conditions** |  |  |  |  |  |  |  |  |
| Enthesopathies & synovial disorders | 12.42% | 0.66% | 1.03% | 10.74% |  | 5.28% | 8.29% | 86.44% |
| Osteoarthritis (excl spine) | 9.16% | 1.94% | 2.89% | 4.33% |  | 21.20% | 31.54% | 47.26% |
| Spondylosis | 2.58% | 0.72% | 0.29% | 1.56% |  | 28.02% | 11.38% | 60.60% |
| Gout | 2.20% | 0.13% | 0.37% | 1.70% |  | 6.03% | 16.76% | 77.22% |
| Intervertebral disc disorders | 2.18% | 0.77% | 0.46% | 0.95% |  | 35.17% | 21.17% | 43.67% |
| Osteoporosis | 2.16% | 0.33% | 0.55% | 1.29% |  | 15.05% | 25.42% | 59.52% |
| Rheumatoid arthritis | 0.86% | 0.28% | 0.33% | 0.25% |  | 32.41% | 38.36% | 29.23% |
| Spinal stenosis | 0.74% | 0.32% | 0.18% | 0.24% |  | 43.32% | 24.53% | 32.15% |
| Fibromatoses | 0.69% | 0.03% | 0.20% | 0.46% |  | 4.16% | 28.44% | 67.40% |
| Polymyalgia rheumatica | 0.53% | 0.05% | 0.18% | 0.30% |  | 9.91% | 34.32% | 55.78% |
| Spondylolisthesis | 0.25% | 0.12% | 0.04% | 0.09% |  | 47.02% | 16.09% | 36.88% |
| Collapsed vertebra | 0.23% | 0.05% | 0.02% | 0.15% |  | 23.76% | 10.74% | 65.50% |
| Psoriatic arthritis | 0.20% | 0.02% | 0.06% | 0.12% |  | 10.10% | 31.33% | 58.57% |
| Ankylosing spondylitis | 0.13% | 0.03% | 0.04% | 0.06% |  | 26.12% | 28.87% | 45.00% |
| Lupus erythematosus | 0.12% | 0.02% | 0.05% | 0.05% |  | 20.80% | 39.87% | 39.35% |
| Giant cell arteritis | 0.10% | 0.01% | 0.04% | 0.05% |  | 14.24% | 37.80% | 47.96% |
| Sjogren's syndrome | 0.09% | 0.02% | 0.03% | 0.04% |  | 22.71% | 31.97% | 45.32% |
| Scleroderma | 0.03% | 0.01% | 0.01% | 0.01% |  | 18.06% | 41.80% | 40.15% |
| Enteropathic arthropathy | 0.01% | 0.01% | 0.00% | 0.00% |  | 60.32% | 9.05% | 30.54% |
|  |  |  |  |  |  |  |  |  |
| **Neurological conditions** |  |  |  |  |  |  |  |  |
| Migraine | 4.40% | 0.24% | 0.34% | 3.82% |  | 5.43% | 7.72% | 86.86% |
| Peripheral neuropathy | 1.55% | 0.31% | 0.19% | 1.05% |  | 19.71% | 12.48% | 67.82% |
| Epilepsy | 1.25% | 0.24% | 0.57% | 0.44% |  | 19.26% | 45.42% | 35.32% |
| Chronic fatigue syndrome | 0.92% | 0.09% | 0.22% | 0.61% |  | 9.38% | 23.79% | 66.84% |
| Trigeminal neuralgia | 0.30% | 0.01% | 0.03% | 0.25% |  | 5.01% | 11.61% | 83.38% |
| Diabetic neuropathy | 0.26% | 0.07% | 0.04% | 0.15% |  | 26.17% | 15.21% | 58.61% |
| Parkinson's disease | 0.23% | 0.04% | 0.11% | 0.07% |  | 17.74% | 50.19% | 32.07% |
| Essential tremor | 0.22% | 0.02% | 0.02% | 0.18% |  | 8.18% | 9.34% | 82.47% |
| Multiple sclerosis | 0.20% | 0.03% | 0.11% | 0.06% |  | 13.85% | 56.60% | 29.55% |
| Autonomic neuropathy | 0.14% | 0.05% | 0.02% | 0.07% |  | 36.30% | 13.31% | 50.39% |
| Cerebral palsy | 0.13% | 0.05% | 0.05% | 0.03% |  | 41.54% | 38.22% | 20.25% |
| Intracranial hypertension | 0.05% | 0.02% | 0.02% | 0.01% |  | 34.04% | 46.88% | 19.07% |
| Myasthenia gravis | 0.03% | 0.00% | 0.02% | 0.01% |  | 10.94% | 53.82% | 35.23% |
| Motor neuron disease | 0.02% | 0.01% | 0.00% | 0.00% |  | 33.93% | 33.20% | 32.80% |
|  |  |  |  |  |  |  |  |  |
| **Perinatal conditions** |  |  |  |  |  |  |  |  |
| Congenital septal defect | 0.31% | 0.10% | 0.11% | 0.10% |  | 32.20% | 34.49% | 33.31% |
| Down's syndrome | 0.06% | 0.01% | 0.03% | 0.01% |  | 25.00% | 55.16% | 19.83% |
| Spina bifida | 0.06% | 0.03% | 0.01% | 0.02% |  | 48.14% | 25.13% | 26.71% |
|  |  |  |  |  |  |  |  |  |
| **Skin conditions** |  |  |  |  |  |  |  |  |
| Dermatitis | 16.97% | 0.30% | 0.59% | 16.08% |  | 1.77% | 3.45% | 94.78% |
| Acne | 5.52% | 0.01% | 0.02% | 5.50% |  | 0.13% | 0.28% | 99.59% |
| Seborrheic dermatitis | 2.51% | 0.01% | 0.01% | 2.49% |  | 0.45% | 0.23% | 99.32% |
| Psoriasis | 2.31% | 0.08% | 0.26% | 1.98% |  | 3.43% | 11.11% | 85.46% |
| Rosacea | 1.55% | 0.01% | 0.02% | 1.53% |  | 0.40% | 1.13% | 98.47% |
| Alopecia areata | 0.32% | 0.00% | 0.00% | 0.31% |  | 0.77% | 0.84% | 98.39% |
| Hidradenitis suppurativa | 0.26% | 0.01% | 0.03% | 0.22% |  | 4.36% | 11.87% | 83.77% |
| Vitiligo | 0.24% | 0.01% | 0.01% | 0.23% |  | 2.60% | 2.32% | 95.08% |

# **Additional file Table S3 – Coefficients of ethnic categories estimated from regressions on probability of patients having a diagnosis recorded in different settings.**

|  | **Probability of diagnosis recorded in different settings** | | | |
| --- | --- | --- | --- | --- |
|  | **(1)** | **(2)** | **(3)** | **(4)** |
|  | **secondary care only** | **secondary and primary care** | **primary care only** | **secondary or primary care** |
| **Regression with higher-level ethnic categories** | | |  |  |
| White (reference group) | |  |  |  |
| Asian | -0.0001*** | -0.0002*** | -0.0006*** | -0.0009*** |
|  | (-20.085) | (-34.563) | (-61.016) | (-57.117) |
| Black | -0.0003*** | -0.0003*** | -0.0002*** | -0.0008*** |
|  | (-35.637) | (-35.682) | (-15.854) | (-39.016) |
| Mixed | -0.0003*** | -0.0004*** | -0.0006*** | -0.0013*** |
|  | (-32.200) | (-43.310) | (-37.514) | (-51.965) |
| Other | -0.0007*** | -0.0010*** | -0.0014*** | -0.0031*** |
|  | (-67.023) | (-90.325) | (-82.639) | (-111.317) |
| Unknown | -0.0014*** | -0.0019*** | -0.0024*** | -0.0057*** |
|  | (-328.114) | (-390.955) | (-323.231) | (-461.581) |
|  |  |  |  |  |
| **Regression with lower-level ethnic categories** | | |  |  |
| White (reference group) |  |  |  |  |
| Indian | -0.0001*** | -0.0001*** | -0.0003*** | -0.0004*** |
|  | (-4.5318) | (-6.5804) | (-19.5779) | (-15.5385) |
| Pakistani | 0.0003*** | 0.0003*** | 0.0004*** | 0.0010*** |
|  | (22.2733) | (17.2923) | (22.1900) | (28.3830) |
| Bangladeshi | 0.0003*** | 0.0003*** | 0.0008*** | 0.0014*** |
|  | (16.9653) | (14.9696) | (26.9970) | (28.1343) |
| Chinese | -0.0010*** | -0.0014*** | -0.0033*** | -0.0056*** |
|  | (-90.5173) | (-116.3686) | (-178.6191) | (-185.3948) |
| Other Asian | -0.0005*** | -0.0007*** | -0.0014*** | -0.0026*** |
|  | (-47.3915) | (-61.3745) | (-79.9657) | (-89.7858) |
| Unknown Asian | 0.0002*** | 0.0001*** | 0.0004*** | 0.0008*** |
|  | (12.7034) | (8.4556) | (17.3806) | (18.8076) |
| Caribbean | 0.0001*** | 0.0004*** | 0.0008*** | 0.0013*** |
|  | (6.3534) | (15.8920) | (31.7348) | (27.6640) |
| African | -0.0005*** | -0.0006*** | -0.0007*** | -0.0019*** |
|  | (-57.3454) | (-62.4193) | (-46.6570) | (-76.5821) |
| Other Black | -0.0003*** | -0.0005*** | -0.0006*** | -0.0013*** |
|  | (-12.3073) | (-22.9432) | (-19.0296) | (-25.4647) |
| Unknown Black | -0.0001*** | -0.0002*** | 0.0002*** | -0.0001** |
|  | (-8.5561) | (-9.9499) | (8.6145) | (-2.6412) |
| Mixed | -0.0003*** | -0.0004*** | -0.0005*** | -0.0012*** |
|  | (-31.7028) | (-42.7837) | (-36.6454) | (-51.0485) |
| Other | -0.0007*** | -0.0010*** | -0.0014*** | -0.0031*** |
|  | (-66.8863) | (-90.1847) | (-82.2902) | (-111.0058) |
| Unknown | -0.0014*** | -0.0019*** | -0.0024*** | -0.0057*** |
|  | (-327.6340) | (-390.5011) | (-322.3665) | (-460.7712) |

Note: The models include interaction between age and gender, newly registered patients, condition fixed effects and GP fixed effects. * p<0.05 ** p<0.01 *** p<0.001, t-statistic in parentheses

# **Additional file Table S4 – Coefficients of deprivation quintiles estimated from regressions on probability of patients having a diagnosis recorded in different settings.**

|  | **Probability of diagnosis recorded in different settings** | | | |
| --- | --- | --- | --- | --- |
|  | **(1)** | **(2)** | **(3)** | **(4)** |
|  | **secondary care only** | **secondary and primary care** | **primary care only** | **secondary or primary care** |
| Patient area-level Index of Multiple Deprivation | | |  |  |
| 1 – least deprived (reference group) | |  |  |  |
| 2 | 0.0002*** | 0.0002*** | 0.0002*** | 0.0006*** |
|  | (24.959) | (30.963) | (17.894) | (34.365) |
| 3 | 0.0003*** | 0.0004*** | 0.0003*** | 0.0011*** |
|  | (44.530) | (54.619) | (34.335) | (62.332) |
| 4 | 0.0005*** | 0.0007*** | 0.0006*** | 0.0018*** |
|  | (66.694) | (80.140) | (57.977) | (96.197) |
| 5 – most deprived | 0.0007*** | 0.0010*** | 0.0010*** | 0.0027*** |
|  | (90.172) | (106.039) | (84.506) | (132.310) |
| Unknown | 0.0004*** | 0.0005*** | 0.0005*** | 0.0014*** |
|  | (8.043) | (8.684) | (7.306) | (11.418) |

Note: The models include interaction between age and gender, newly registered patients, condition fixed effects and GP fixed effects. * p<0.05 ** p<0.01 *** p<0.001, t-statistic in parentheses

# **Additional file Figure S1 – Figure 4. Inequalities in GP practice fixed-effects on diagnostic recording by care setting**


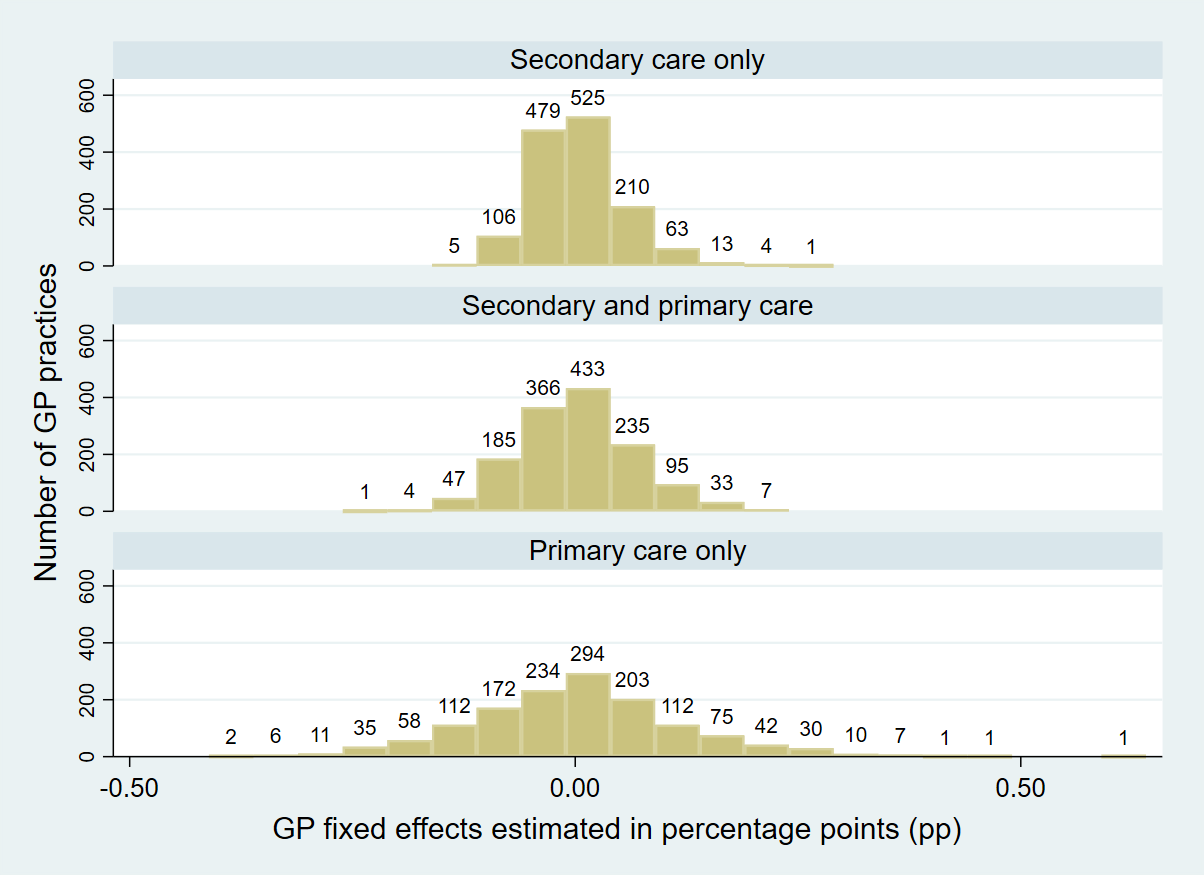


# **Additional file Table S5 – Coefficients of ethnic categories estimated from sensitivity analyses removing GP fixed-effects and including more recent diagnoses**

(1) without control GP fixed effects, and

(2) focused on new diagnoses only by excluding cases recorded before 1 April, 2006.

| **Sensitivity analysis** | 1. **without GP fixed effects** | | |  | 1. **focused on new diagnoses only** | | |
| --- | --- | --- | --- | --- | --- | --- | --- |
|  | **(1)** | **(2)** | **(3)** |  | **(1)** | **(2)** | **(3)** |
|  | **secondary care**  **only** | **secondary and**  **primary care** | **primary care**  **only** |  | **secondary care**  **only** | **secondary and**  **primary care** | **primary care**  **only** |
| **Ethnicity** |  |  |  |  |  |  |  |
| White (reference group) | |  |  |  |  |  |  |
| Mixed | -0.0003*** | -0.0004*** | -0.0006*** |  | -0.0003*** | -0.0004*** | -0.0008*** |
|  | (-28.562) | (-45.628) | (-40.130) |  | (-37.330) | (-46.659) | (-48.973) |
| Asian | -0.0000 | -0.0002*** | -0.0005*** |  | -0.0002*** | -0.0003*** | -0.0009*** |
|  | (-1.485) | (-23.486) | (-59.482) |  | (-31.690) | (-47.305) | (-91.775) |
| Black | -0.0001*** | -0.0002*** | -0.0001*** |  | -0.0003*** | -0.0003*** | -0.0005*** |
|  | (-16.362) | (-28.946) | (-10.278) |  | (-40.447) | (-43.962) | (-38.609) |
| Other | -0.0006*** | -0.0010*** | -0.0014*** |  | -0.0008*** | -0.0009*** | -0.0019*** |
|  | (-60.452) | (-94.374) | (-85.950) |  | (-79.793) | (-97.352) | (-104.505) |
| Unknown | -0.0014*** | -0.0020*** | -0.0025*** |  | -0.0015*** | -0.0018*** | -0.0031*** |
|  | (-334.708) | (-408.048) | (-334.139) |  | (-354.340) | (-401.411) | (-386.786) |

| **Sensitivity analysis** | **1. without GP fixed effects** | | |  | **2. focused on new diagnoses only** | | |
| --- | --- | --- | --- | --- | --- | --- | --- |
|  | **(1)** | **(2)** | **(3)** |  | **(1)** | **(2)** | **(3)** |
|  | **secondary care**  **only** | **secondary and**  **primary care** | **primary care**  **only** |  | **secondary care**  **only** | **secondary and**  **primary care** | **primary care**  **only** |
| **Patient area-level Index of Multiple Deprivation** | | | |  |  |  |  |
| 1 – least deprived (reference group) | |  |  |  |  |  |  |
| 2 | 0.0002*** | 0.0003*** | 0.0002*** |  | 0.0002*** | 0.0002*** | 0.0002*** |
|  | (30.234) | (40.695) | (18.160) |  | (24.230) | (27.840) | (21.476) |
| 3 | 0.0003*** | 0.0004*** | 0.0003*** |  | 0.0003*** | 0.0003*** | 0.0004*** |
|  | (53.029) | (64.105) | (29.716) |  | (43.564) | (49.294) | (41.609) |
| 4 | 0.0005*** | 0.0007*** | 0.0005*** |  | 0.0005*** | 0.0005*** | 0.0008*** |
|  | (85.749) | (104.617) | (64.413) |  | (66.456) | (73.826) | (68.263) |
| 5 – most deprived | 0.0009*** | 0.0012*** | 0.0012*** |  | 0.0007*** | 0.0008*** | 0.0012*** |
|  | (138.153) | (171.502) | (131.919) |  | (90.908) | (99.273) | (97.682) |
| Unknown | 0.0003*** | 0.0005*** | 0.0006*** |  | 0.0004*** | 0.0004*** | 0.0006*** |
|  | (6.697) | (8.694) | (7.961) |  | (7.819) | (8.322) | (8.485) |

Note: The models include interaction between age and gender, newly registered patients, disease fixed effects and GP fixed effects. * p<0.05 ** p<0.01 *** p<0.001, t-statistic in parentheses

1. [↑](#footnote-ref-1)
